# Acoustic-optical phonon up-conversion and hot-phonon bottleneck in lead-halide perovskites

**DOI:** 10.1038/ncomms14120

**Published:** 2017-01-20

**Authors:** Jianfeng Yang, Xiaoming Wen, Hongze Xia, Rui Sheng, Qingshan Ma, Jincheol Kim, Patrick Tapping, Takaaki Harada, Tak W. Kee, Fuzhi Huang, Yi-Bing Cheng, Martin Green, Anita Ho-Baillie, Shujuan Huang, Santosh Shrestha, Robert Patterson, Gavin Conibeer

**Affiliations:** 1 Australian Centre for Advanced Photovoltaics, School of Photovoltaics and Renewable Energy Engineering, University of New South Wales, Sydney, New South Wales 2052, Australia; 2 Centre for Micro-Photonics, Swinburne University of Technology, Melbourne, Victoria 3122, Australia; 3 Department of Chemistry, The University of Adelaide, Adelaide, South Australia 5005, Australia; 4 State Key Lab of Advanced Technologies for Materials Synthesis and Processing, Wuhan University of Technology, Wuhan 430070, China; 5 Department of Materials Science and Engineering, Monash University, Melbourne, Victoria 3800, Australia

## Abstract

The hot-phonon bottleneck effect in lead-halide perovskites (APbX_3_) prolongs the cooling period of hot charge carriers, an effect that could be used in the next-generation photovoltaics devices. Using ultrafast optical characterization and first-principle calculations, four kinds of lead-halide perovskites (A=FA^+^/MA^+^/Cs^+^, X=I^−^/Br^−^) are compared in this study to reveal the carrier-phonon dynamics within. Here we show a stronger phonon bottleneck effect in hybrid perovskites than in their inorganic counterparts. Compared with the caesium-based system, a 10 times slower carrier-phonon relaxation rate is observed in FAPbI_3_. The up-conversion of low-energy phonons is proposed to be responsible for the bottleneck effect. The presence of organic cations introduces overlapping phonon branches and facilitates the up-transition of low-energy modes. The blocking of phonon propagation associated with an ultralow thermal conductivity of the material also increases the overall up-conversion efficiency. This result also suggests a new and general method for achieving long-lived hot carriers in materials.

## Abstract (web summary)

Slow cooling of hot charge carriers in lead halide perovskite could be used in photovoltaics devices. Here, Yang *et al*. study hot carrier dynamics by transient absorption spectroscopy. They relate the phonon bottleneck to the up-conversion of low-energy phonons, facilitated by the presence of organic cations.

Significant progress has been achieved in metal-halide perovskite (APbX_3_) solar cells with the certified efficiency record now exceeding 20%, just a few years after the first solid-state device was reported to have an efficiency of 9.7% in 2012 (refs 1, 2, 3, 4, 5). The photophysics and carrier dynamics in lead-halide perovskites have been intensively studied in the last few years because of their promising performance in the conventional photovoltaic devices. The rapid emergence of lead-halide perovskites is attributed to their outstanding optoelectronic properties, including superb optical absorption, high ambipolar charge mobility and appropriate band gap^6^,^7^. Recently, a significant hot-phonon bottleneck effect in carrier thermalization was also observed in lead-halide perovskites^8^,^9^, which indicates potential applications of these kinds of materials in the advanced concept of hot carrier optoelectronics.

The so-called hot-phonon bottleneck effect denotes a phenomenon where the relaxation rate of a non-equilibrium carrier-phonon system is reduced when the carrier injection level is high^10^,^11^,^12^,^13^,^14^. Cooling of photo-generated hot carriers in a material dissipates the absorbed optical energy as lattice heat via longitudinal optical (LO) phonon emission and decay. Thermalization by this mechanism leads to about 50% of the energy losses in a traditional single junction solar cell^15^. A strong phonon bottleneck effect is helpful to establish a long-lived hot carrier population, which is critical to achieve a working hot carrier photovoltaic device^16^,^17^,^18^ and break the Shockley–Queisser limit^19^ for photovoltaic energy conversion. Hot carrier properties of materials are also important in photodetection^20^, photocatalysis^21^ and light emission^22^,^23^ to improve the power efficiency and carrier dynamics, especially in high-power optoelectronic applications such as lasing^24^,^25^,^26^.

Although several mechanisms have been suggested for the hot carrier dynamics on sub-picosecond timescales in lead-halide perovskites^27^,^28^,^29^, a comprehensive understanding of the phonon bottleneck effect occurring on longer timescales (up to thousand picoseconds) is still lacking. Slow carrier cooling and the bottleneck phenomenon can be caused by different mechanisms, such as a modified carrier-phonon interaction and a blocked hot-phonon decay in general. However, no clear conclusion has been made in previous studies for lead-halide perovskites.

In this study, we attempt to provide further insight on the phonon bottleneck effect in the lead-halide perovskite family. We compare the hot carrier and phononic properties among four typical kinds of lead-halide perovskites used in photovoltaics, including CH_3_NH_3_PbI_3_ (MAPbI_3_), HC(NH_2_)_2_PbI_3_ (FAPbI_3_), CH_3_NH_3_PbBr_3_ (MAPbBr_3_) and CsPbIBr_2_. By varying the A-site cations (MA^+^, FA^+^, Cs^+^) and X-site halide anions (I^−^, Br^−^), we try to elucidate the impact of lattice composition on the carrier cooling properties. A complete picture of hot carrier relaxation in lead-halide perovskites is presented using the ultrafast transient absorption (TA) technique. Further studies of the phonon band structures and emission rates reveal the dynamics behind the relaxation. The phonon bottleneck effect in these materials is explained by acoustic phonon up-conversion, which leads to a LO phonon emission rate that is 10 times slower in FAPbI_3_ compared with the inorganic counterpart under the same experimental conditions. The presence of rotatable groups on the organic sub-lattice is proposed to be the essential reason for this efficient up-transition dynamics. To the best of our knowledge, this mechanism has been ignored previously in lead-halide perovskite research and is also rarely discussed by the hot carrier research community. This study connects a variety of material properties in lead-halide perovskites together. The proposed mechanism is also enlightening for the future exploration of new functional hot carrier materials.

## Results

### Power-dependent carrier temperature decay

All the samples used in this study, including MAPbI_3_, FAPbI_3_, MAPbBr_3_ and CsPbIBr_2_, were prepared on glass substrates with a thickness about 300 nm. The sample preparations are described in the Methods section and the detailed characterizations have been reported elsewhere^30^,^31^,^32^,^33^. The fundamental absorption band gaps were determined to be 1.69, 1.55, 2.31 and 2.05 eV for the MAPbI_3_, FAPbI_3_, MAPbBr_3_ and CsPbIBr_2_ samples, respectively. On the basis of the fabrication methods, all samples are optimized in conventional photovoltaic applications, which are also sufficiently stable during the measurements to ensure that accurate and reproducible experimental data are obtained. All the femtosecond pump-probe TA measurements (see ‘Methods' section) were conducted under identical ambient conditions at room temperature (295 K). A pump pulse with a wavelength of 400 nm and duration of 100 fs was used to excite carriers well above the band gap for all the samples, while an ultrashort broadband super-continuum laser covering the whole visible range was used as the probe. The pump-induced change in the absorption spectrum was acquired as a function of time delay. The carrier relaxation dynamics were then resolved via the bleaching in the spectra above the band edge under different excitation fluences.

**Figure 1:**
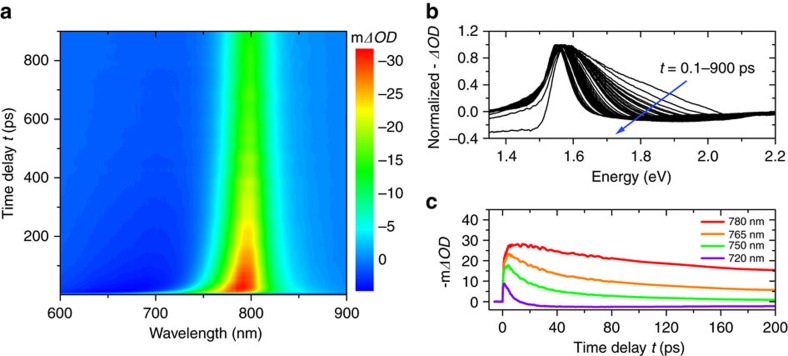
Transient absorption spectra of the FAPbI_3_ sample. (**a**) 3D TA spectrum pumped at 400 nm with an initial carrier concentration *N*_0_=4.84 × 10^18^ cm^−3^. The initial broadening of the bleaching signal extending above the apparent band gap energy indicates the temporary presence of hot carriers. (**b**) Normalized negative TA spectra (−*ΔOD*) at different pump-probe time delays *t* from 0.1 to about 900 ps. (**c**) Decay of the bleaching signal given as minus milli *ΔOD* (−m*ΔOD*) at different wavelengths showing carrier relaxation kinetics.

Figure 1 shows the TA spectrum obtained from the FAPbI_3_ sample under a pump fluence of 90 μJ cm^−2^ with an initial carrier concentration around *N*_0_=4.84 × 10^18^ cm^−3^ (see Supplementary Methods for carrier injection estimation). Sustained absorption bleaching is peaked around the apparent band gap (about 1.55 eV) and broadening into the high-energy region, whose gradual narrowing as shown in Fig. 1b,c exhibits the carrier thermalization process. The so-called phonon bottleneck effect is intuitively demonstrated by a power-dependent measurement. The bleaching intensity in the high-energy region is markedly reduced under a pump fluence of 10 μJ cm^−2^, indicating a variation in carrier relaxation dynamics under different concentrations (Supplementary Fig. 1). Under similar experimental conditions, power-dependent TA measurements were also performed on the other three samples. The measured 3D TA spectra under the maximum pump fluence (about 100 μJ cm^−2^) in each sample are given in Supplementary Fig. 2.

**Figure 2:**
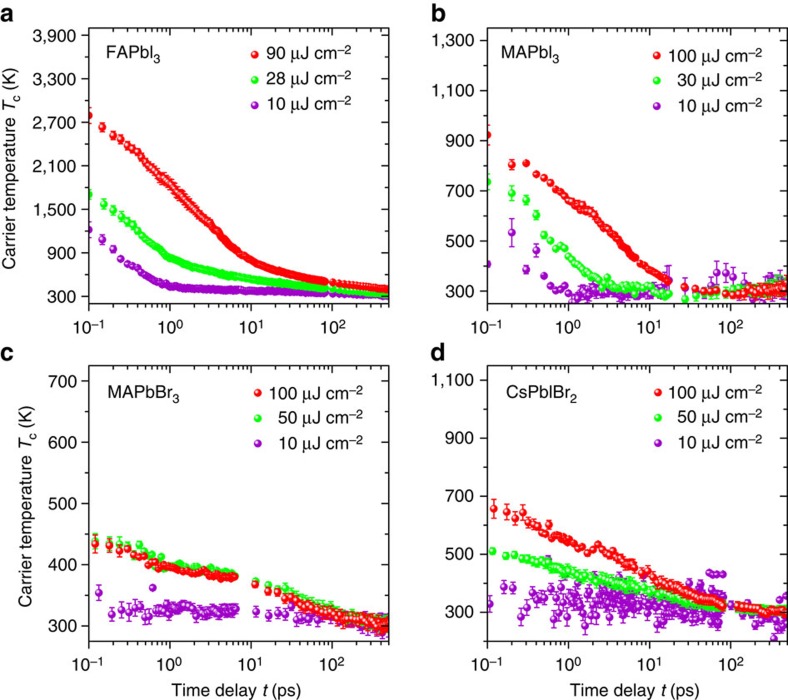
Time-dependent carrier temperature under different incident fluence. In (**a**) FAPbI_3_, (**b**) MAPbI_3_, (**c**) MAPbBr_3_, (**d**) CsPbIBr_2_. The carrier temperatures are extracted by fitting the high-energy tail of the bleaching in the TA spectra. The error bar shows the standard error of the average (s.e.m.) fitting results.

The carrier temperature *T*_c_ is obtained by fitting the high-energy tail of the bleaching spectrum above the band edge^34^,^35^,^36^ whose line shape is approximated to be a modified Maxwell–Boltzmann distribution (see Supplementary Methods for carrier temperature fitting section 1). The fittings are then conducted among all the measured TA spectra following the same tail selection scheme with a careful sensitivity analysis to ensure reliable and comparable fitting results. The fitted carrier temperatures as a function of time delay *t* are summarized in Fig. 2 (see Supplementary Methods section 2 and section 3 for carrier temperature fitting).

Among different lead-perovskite samples, two primary features are shared according to their cooling curves. First, all the samples show an evident temperature-dependent carrier cooling rate, which is apparently decreased when the carrier temperature is lower. Although the primary carrier temperature decrease roughly occurs within the first 10 ps in these samples, a subsequent slower cooling rate extends the overall relaxation period up to several hundred picoseconds. On the same cooling curve of FAPbI_3_ under 90 μJ cm^−2^ fluence in Fig. 2a, an average cooling lifetime of 25 and 124 ps are observed beginning at *T*_c_=1,500 K and 700 K, respectively. These average lifetimes are obtained using a double-exponential fitting, which is applied from the selected initial high carrier temperatures to the end of the cooling curve.

Second, two stages of the carrier cooling process are identified from Fig. 2, whose cooling rates exhibit a different power dependence. The first cooling stage shows a very similar sub-picosecond lifetime under different excitation intensities. This stage is dominated by the intrinsic Fröhlich phonon emission corresponding to the typical timescales studied before in lead-halide perovskites^37^. A limited thermalization rate for hot holes is observed in this stage compared with conventional organic semiconductors. A reduced electronic density of state (DOS) near the fundamental valence band maximum was used to explain the restricted relaxation of carriers, which leads to a nearly power-independent cooling rate observed here. Since the electronic band structure near the band edge is primarily dominated by the lead-halide bond^37^,^38^, similar cooling lifetimes and their power independence are also observed in the inorganic CsPbIBr_2_ sample on this stage. The second cooling stage is then observed in the later period under a sufficiently high fluence (larger than 10 μJ cm^−2^ here). The cooling lifetime increases with a larger carrier density in the second stage evidently, corresponding to the effect of a phonon bottleneck. Just following the similar double-exponential fitting used above from a fixed initial temperature *T*_c_=700 K, the cooling lifetime of FAPbI_3_ grows from 45 to 124 ps when the fluence is tuned from 28 to 90 μJ cm^−2^ correspondingly.

Compared with FAPbI_3_, an average cooling lifetime of only 6 ps beginning from *T*_c_=700 K is observed in our MAPbI_3_ sample under the maximum pumping with a much lower initial carrier temperature. This observation is inconsistent with the previous theoretical and experimental conclusion on MAPbI_3_, although a power-dependent bottleneck effect is also observed in its second cooling stage. The changes in the organic cation (FA^+^ and MA^+^) with the same kind of lead-halide framework should not strongly perturb both the electronic and phononic properties of the material^8^,^27^,^37^,^38^. This significant difference is believed to be caused by the lattice defects. Our previous observation confirms that the MAPbI_3_ sample prepared by the gas-assisted method (see ‘Methods' section) has a large density of sub-band-gap trapping states^39^. The fast carrier relaxation observed here convinces us that a large number of above-band-gap defects are also presented in this sample, which could provide more relaxation pathways for the hot carriers^40^. It is noted that the solar cell made of such a material can still achieve a fairly good energy conversion efficiency up to 17% (ref. 30). This suggests the relaxation of hot carriers is more sensitive to the presence of lattice defeats, so special attention to material quality is required.

Although the initial carrier temperatures in MAPbBr_3_ and CsPbIBr_2_ are lower than that in FAPbI_3_ and MAPbI_3_ due to larger band gaps, power-dependent cooling lifetimes are still observed in their second cooling stage. Under a similar carrier concentration, the bottleneck effect between all the materials can be compared by investigating the second cooling stage in a similar carrier temperature range. To intuitively show the difference in the bottleneck effect among these samples, we first compare their average cooling lifetimes fitted below the same carrier temperature *T*_c_=400 K for an intermediate fluence (28 μJ cm^−2^ for FAPbI_3_, 50 μJ cm^−2^ for MAPbBr_3_ and CsPbIBr_2_). All the samples are then in the second cooling stage with a similar carrier density of about *N*_0_=2 × 10^18^ cm^−3^ for ready comparison. A cooling lifetime of about 305 ps is observed in the FAPbI_3_ sample in this period, while only a lifetime of 71 and 37 ps are observed in the MAPbBr_3_ and CsPbIBr_2_ sample, respectively. When the excitation intensity increases to the maximum one used in our measurements with a carrier density of about *N*_0_=5 × 10^18^ cm^−3^, the cooling lifetime of FAPbI_3_ is significantly increased to around 1 ns after *T*_c_=400 K while only about 48 ps is achieved in CsPbIBr_2_.

An unusual saturation effect was observed in the MAPbBr_3_ sample (Fig. 2c), where both the TA spectra and carrier temperature show nearly the same result when the excitation is larger than 50 μJ cm^−2^, corresponding to a carrier density about *N*_0_=2.69 × 10^18^ cm^−3^. Since there is no evidence of either saturation in absorption at the pumping wavelength (400 nm) or a lasing effect in MAPbBr_3_ according to the TA spectrum, we consider this observation to be a result of Auger-Impact Ionization (AI) processes^41^. It has been reported that the Auger coefficient in MAPbBr_3_ is about four times larger than that in MAPbI_3_ (ref. 42). Triggered by a sufficiently high excess carrier concentration, it is possible that a strong multi-particle interaction occurs in this material, limiting the change of carrier temperature under a higher excitation power density.

### Phonon relaxation and bottleneck effect

To understand the mechanism behind the bottleneck effect observed in lead-halide perovskites, we first review some general understandings of carrier-phonon dynamics in solid-state polar semiconductors. The carrier thermalization is accompanied by the emission of LO phonons, which then decay to the low-energy acoustical modes and lead to local lattice heating. With an effective thermal transfer to the surroundings via acoustic phonon propagation, the carrier's excess kinetic energy is eventually dissipated in an irreversible manner. Therefore, three distinct relaxation stages exist: (1) carrier-phonon scattering (Fröhlich interaction); (2) optical phonon decay to acoustic phonons; (3) acoustic phonon propagation to the far-field region in the material (thermal conduction). Correspondingly, in a non-quantized system with continuous electronic DOS, possible blocking mechanisms can be introduced in each relaxation stage to prolong the overall cooling period of hot carriers: (1) thermal isolation between the optical phonon and carrier population; (2) reducing the phononic DOS; (3) acoustic phonon up-conversion to optical phonon.

Strong thermal isolation required by the first mechanism is very difficult to achieve due to the spontaneous interaction between the carriers and LO phonons. In lead-halide perovskites, the Fröhlich interaction mainly occurs between the charge carriers and lead-halide bond^27^,^37^, which governs the carrier relaxation in the first cooling stage. Although the large polaron effect proposed recently^29^ demonstrates that a reduced carrier-phonon coupling may exist in the organic–inorganic lead-halide perovskites, a Fröhlich scattering rate similar to the typical III–V semiconductor materials, such as GaAs, has also been reported^8^,^27^. In particular, since the electron-phonon coupling occurs constantly during carrier thermalization, just a change of Fröhlich coupling also cannot explain why a power-dependent carrier cooling rate is only observed in the second cooling stage while the rate in the first stage remains nearly unaffected. Therefore, it can be ruled out as a major cause of the phonon bottleneck effect here. The second method is one of the major mechanisms utilized in the current carrier thermalization engineering. In typical semiconductor systems, the decay mechanisms of LO phonon to low-energy acoustic modes include the symmetric Klemens-decay and anti-symmetric Ridley-decay^17^. Opening the phonon band gap (between the optical and acoustical branches) using compounds with a large atomic mass-difference has been intensively studied before^17^,^18^,^43^,^44^ to slow carrier cooling, since the Klemens-decay mechanism is blocked if the phonon band gap is greater than twice of the highest acoustic phonon energy. The third blocking mechanism happens in the last stage of carrier cooling where the induced local lattice heat finally dissipates to the surroundings. An efficient acoustic phonon up-conversion can recycle the thermal energy back and reheat carriers^10^.

**Figure 3:**
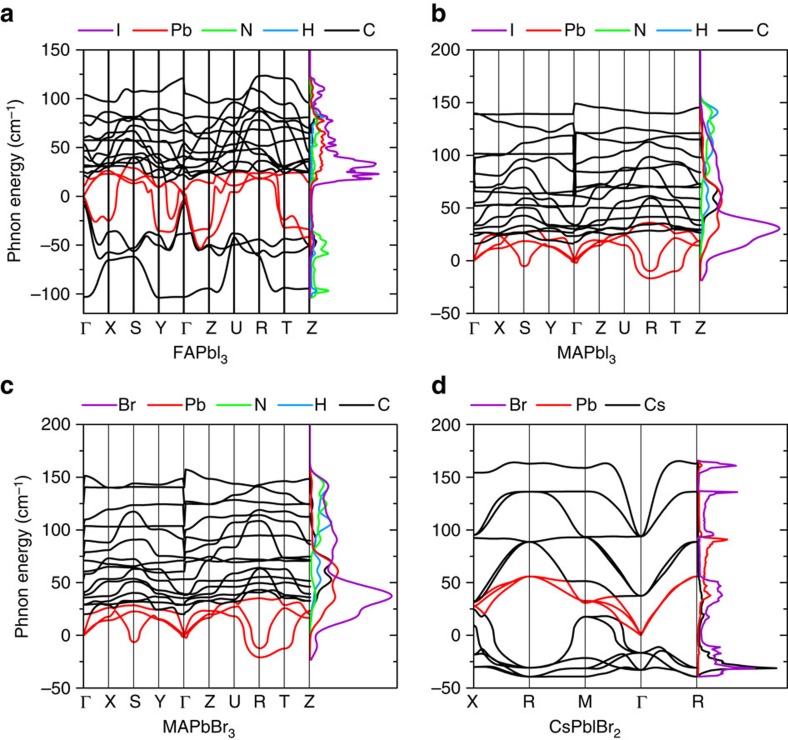
Phonon band structure and projected phonon DOS. In (**a**) FAPbI_3_; (**b**) MAPbI_3_; (**c**) MAPbBr_3_; (**d**) CsPbBr_3_. The projected phonon DOS are given in arbitrary units (a.u.) and show the contributions of the organic cation and inorganic sub-lattice respectively. The acoustic bands are plotted in red.

With this background knowledge in mind, we first compare the phonon band structures of these materials. The phonon band structures of FAPbI_3_, MAPbI_3_, MAPbBr_3_ and CsPbBr_3_ (as an analogue of CsPbIBr_2_) were calculated from first-principles using density functional theory (see ‘Methods' section). All the structures were optimized based on a pseudocubic lattice, which has been extensively used to predict properties for these materials^45^,^46^,^47^. The calculation results have also been compared with previous reports to ensure a good estimation of the phonon energy distribution. Figure 3 shows the calculated phonon band structures (below 200 cm^−1^). All the acoustic bands are plotted as red curves. The imaginary phonon modes with a negative frequency result from the metastable lattice of the perovskite where the permanent displacement of the atoms is possible through distortions of the crystal lattice^37^,^48^. The projected density of states on each atom is also given in Fig. 3 to show the detailed contributions from each sub-lattice.

Evidently, no complete phonon band gap is observed in these four kinds of perovskite, therefore, there is no significant blocking effect from the second mechanism mentioned above. Compared with the inorganic type CsPbBr_3_, lattice vibration properties in the hybrid types are strongly altered by the presence of organic cations according to Fig. 3. Different to Cs^+^, whose vibrational modes are primarily distributed over the acoustic branches in CsPbBr_3_, the phonon modes relating to the organic cations show an optical-like character with a good overlap to the lead-halide optical modes in the range roughly from 30 to 100 cm^−1^. This result is consistent with the previous Raman observations^49^ on MAPbI_3_ that the libration and torsion of the MA^+^ is coupled with the lead/halide ions and lead to a series of low-energy optical modes in the range of 60–100 cm^−1^. This unique phonon structure implies that although the organic cations do not govern the Fröhlich interaction directly, the overlapping vibration modes can still allow them to participate in the carrier-phonon relaxation through phonon–phonon scattering.

With the influence of non-propagating vibrations on organic cation, most of the optical phonon modes below 200 cm^−1^ in the hybrid types show a relatively flat band dispersion across the first Brillouin zone compared with the inorganic case. A cascade decay of LO phonons may be supported by this mini-band-like structure to slow phonon relaxation. However, it cannot fully explain the much slower carrier cooling lifetime of FAPbI_3_ in the second stage (below *T*_c_=400 K) observed above comparing with MAPbBr_3_, although a similar mini-band-like optical branches are shown in both of them. A series of high-energy (larger than 200 cm^−1^) quasi-static modes are also observed in the hybrid perovskites, which result from the resonant vibrations of H atoms primarily (Supplementary Fig. 3). However, these high-energy modes are considered to be irrelative to the carrier relaxation.

Since no apparent first and second kind of blocking mechanisms exist in these materials, we infer that a relatively effective up-conversion of acoustic phonons may occur to prolong the relaxation period^10^. Actually, the acoustic phonon up-conversion has been reported recently in the perovskite-like organic–inorganic hybrid multiferroic material^50^. In that material, the existence of organic sub-lattices introduces a series of low-energy ‘hybrid phonon' modes, which are defined as the co-vibrations between the organic and inorganic sub-lattices rather than the modes dominated by either kind of sub-lattice alone, according to ref. 50. These low-energy optical-like ‘hybrid phonon' cannot be excited efficiently by an external perturbation but by phonon–phonon scatterings instead. Their large thermal coupling rate to acoustic phonons can facilitate the up-conversion of the latter one and prolong the overall relaxation period of hot-phonon populations.

We adopt the phonon classification concept here used in ref. 50 and classify the phonons in organic–inorganic perovskites according to the projected density of states plotted in Fig. 3a–c and also Supplementary Fig. 3a–c. As pointed out before, the co-vibrations between the organic cation and lead/halide ions roughly in 30–100 cm^−1^ can be called as ‘hybrid phonons'. Since initial Fröhlich scattering occurs on the lead-halide framework primarily, these low-energy ‘hybrid phonons' are mainly excited by phonon–phonon scattering events and these are very similar to the phonon relaxation process occurring in the multiferroic material discussed in ref. 50. Also, these ‘hybrid phonons' showing very little dispersion at around 30 cm^−1^ have a good overlap with the top of the acoustic branches. This kind of phonon band structure is expected to increase the probability of acoustic phonons scattering to optical states and recycle the vibrational energy, corresponding to a good thermal coupling there. On the basis of this information, we therefore propose that a similar acoustic phonon up-conversion process discussed in ref. 50 may also exist in lead-halide perovskites, especially in the organic–inorganic types. Interestingly, according to the previous comparison, the purely inorganic CsPbIBr_2_ sample with a simpler phonon band structure and reduced phononic DOS do have the fastest carrier relaxation rate in the second cooling stage.

To further quantify the relaxation rate of the phonon system, we estimate the average LO phonon emission lifetime *τ*_ave_ for the three samples with good quality (FAPbI_3_, MAPbBr_3_ and CsPbBr_3_) using equation (1), where *T*_L_ is the lattice temperature^36^. Since the elastic scattering between LO phonons and carriers does not dissipate energy irreversibly, the eventual cooling of the carrier-phonon system is caused by the inelastic decay of optical phonons (exciting acoustic phonons). By considering both the emission and reabsorption process of LO modes, the *τ*_ave_ can be taken as an effective cooling lifetime of carriers interacting with a non-equilibrium phonon population^35^,^36^,^51^.


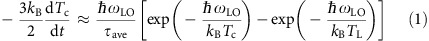


We choose the characteristic/effective LO phonon energies for FAPbI_3_ (ref. 27), MAPbBr_3_ (refs 52, 53), CsPbBr_3_ (refs 54, 55) as 13 meV, 18 meV and 16 meV, respectively, by taking into account both the experimental and theoretical estimations performed before and also the band structure we presented in Fig. 3. These characteristic *ħω*_LO_ are near the top of the optical bands contributed by the lead-halide framework. Complete results for the carrier temperature-dependent LO emission lifetime in each sample are given in Supplementary Fig. 4. All the samples start with a very rapid emission rate *τ*_ave_(*t*)|_*t*→0_≤0.1 ps in the early stage of carrier thermalization without an evident power dependence (Supplementary Fig. 5), since a large non-equilibrium optical phonon population has not yet been achieved in that stage.

**Figure 4:**
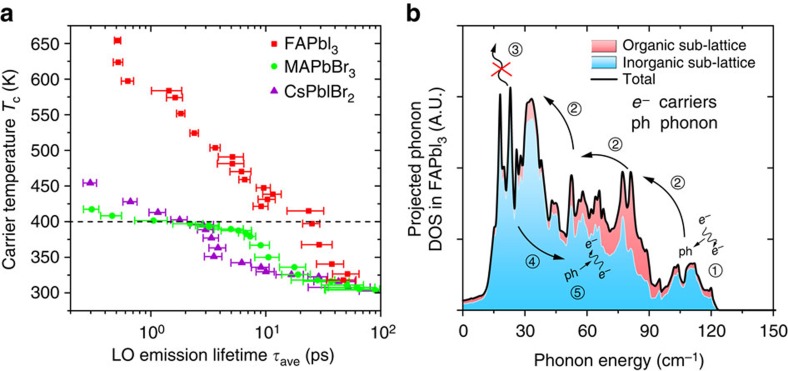
Phonon dynamics and bottleneck effect in lead-halide perovskites. (**a**) Carrier temperature-dependent phonon emission lifetime in different lead-halide perovskites with a similar initial carrier concentration of around 2 × 10^18^ cm^−3^. The error bars shows the standard error of the average (s.e.m) emission lifetime. An emission lifetime that is about 10 times longer than the other materials is observed in FAPbI_3_ with carrier temperature at and below 400 K; (**b**) Proposed phonon dynamics in the FAPbI_3_. The solid black line shows the total phonon DOS, in which the contributions from the inorganic and organic sub-lattices are shown by the blue region on the bottom with the pink region stacked on top, respectively. The labelled phonon dynamic process are: (1) Fröhlich interaction of carriers primarily on the lead-halide framework; (2) relaxation of lead-halide LO phonon, organic sub-lattice can be excited by phonon–phonon scattering; (3) propagation of acoustic phonon is blocked due to anharmonic phonon–phonon scatterings; (4) up-conversion of acoustic phonons; and (5) carrier reheating.

In Fig. 4a, we compare the results from the three perovskite samples with good lattice quality. Again, an intermediate excitation intensity (28 μJ cm^−2^ for FAPbI_3_, 50 μJ cm^−2^ for MAPbBr_3_ and CsPbIBr_2_) is chosen for this comparison to avoid the influence of bleaching saturation in the MAPbBr_3_ sample under a higher pump fluence. We now focus on the second cooling stage in each sample with a carrier temperature around and below 400 K, where the cooling properties are dominated by the phonon bottleneck effect. We find that FAPbI_3_ attains a roughly 10 times slower phonon emission rate than that in CsPbIBr_2_ in this period and around 3 times slower than MAPbBr_3_, as shown in Fig. 4a. A comparison of the LO emission lifetime between FAPbI_3_ and CsPbIBr_2_ under the maximum fluences are also provided in Supplementary Fig. 10. A larger increment of *τ*_ave_ is still observed in FAPbI_3_ leading to an up to 20 times longer emission lifetime than CsPbIBr_2_ around *T*_c_=400 K in that case. Further numerical estimation confirms that this discrepancy does not result from the different LO phonon energies used in the calculation but is dominated by the temperature gradient −d*T*_c_/d*t* among different samples instead. Indeed, the smaller LO phonon energy used for FAPbI_3_ decreases the corresponding emission lifetime a little bit according to equation (1). Combined with the previous cooling lifetime comparison, it suggests that a much stronger bottleneck effect exists in FAPbI_3_ while the pure inorganic counterpart shows the weakest blocking.

The divergence of bottleneck effect observed above could also be explained by different phonon up-conversion efficiencies in these materials, where the proposed up-conversion of acoustic phonon shows a strong correlation to the unique thermal property of lead-halide perovskites. It is expected that the up-conversion of acoustic phonons is accompanied by their rapid and short-range attenuation, which relates microscopically to the thermal conductivity of the material. A strong anharmonic phonon–phonon scattering corresponding to a low thermal conductivity in general can localize acoustic phonons by blocking their propagation and increase the probability of an up-transition. In turn, an efficient up-conversion of acoustic phonon can also impede thermal propagation in the lattice^50^.

In fact, an ultralow thermal conductivity (less than 1 W K^−1^ m^−1^) has been reported in both of the hybrid type lead-halide perovskites^56^,^57^ and their CsPbX_3_ inorganic counterparts^58^. An enhanced anharmonic phonon–phonon scattering resulting from the highly overlapped phonon branches is recognized as the major cause, especially in the hybrid types^57^. The resonant scattering resulting directly from the rotational-like vibrations of organic cation is also contributive^56^,^57^. Both aspects are consistent with the picture of acoustic phonon up-conversion via the low-energy ‘hybrid phonon' modes presented above. In particular, the resonant scattering from the organic cation corresponds to an organic cation-assisted up-conversion route for acoustic phonons, which directly indicates the co-vibrational optical modes can be re-excited by acoustic phonons efficiently in hybrid lead-halide perovskites.

Now back to the divergence of LO emission lifetime in Fig. 4a. Phonon propagation is blocked in CsPbIBr_2_ due to its low thermal conductivity, which increases the phonon up-transition probability. However, the lack of the rotatable organic cation and corresponding altered phonon modes leads to a very limit phonon up-conversion efficiency compared with the other two hybrid types. This suggests the indispensable role of the organic cation, or the large amount of low-energy co-vibrational optical modes, in the phonon up-conversion discussed here. The difference between FAPbI_3_ and MAPbBr_3_ may result from two aspects. On the one hand, a stronger acoustic phonon localization is expected in the FAPbI_3_ compared with MAPbBr_3_. The thermal conductivity of MAPbBr_3_ has been predicted to be higher than its iodide counterparts due to a higher elastic moduli^57^. Practically, the trigonal phase of FAPbI_3_ with a lower lattice-symmetry at room temperature is also expected to further limit its thermal conductivity in general^59^. On the other hand, it looks like a better phonon band overlap near the top of acoustic branches is observed in FAPbI_3_ than those in MAPbBr_3_ according to Fig. 3. This could facilitate the re-excitation of optical modes in the former one more efficiently. Overall, the conclusion is that an efficient up-conversion of low-energy phonons discussed here relies on both the blocking of the acoustic phonons propagation and organic cation-assisted phonon up-transition. Both aspects lead to a significant hot-phonon bottleneck in the hybrid lead-halide perovskites, where the hot-phonon relaxation rate in FAPbI3 is observed as at least 10 times slower than that in the inorganic counterpart via LO phonon emission.

## Discussion

In summary, we propose the following carrier-phonon dynamics in hybrid type lead-halide perovskites to explain the observed strong phonon bottleneck effect under a large carrier population. The proposed process is shown in Fig. 4b. (1) First, the Fröhlich interaction occurs predominantly between the hot carriers and inorganic sub-lattice, exciting the high-energy lead-halide LO phonons; (2) the excited LO phonons then decay to acoustic modes. During this stage, the co-vibration between organic and inorganic sub-lattices can also be excited via phonon–phonon scattering; (3) the propagation of acoustic phonons is blocked due to strong anharmonic phonon–phonon scatterings; (4) the up-transition probability of phonons is then increased, especially when organic cations appear in the lattice. The organic cation introduces lots of low-energy co-vibrational optical modes, which overlap well with acoustic branches and facilitate the phonon up-transition; (5) the recycled thermal (vibrational) energy reheats charge carriers and prolongs the overall cooling period of carrier-phonon system. It is worth emphasizing that processes (3)–(5) are inefficient in conventional semiconductors whose acoustic phonons can easily propagate away along with the hot carrier cooling. The overall thermal recycling will be more efficient when the phonon density is larger since the probability of phonon–phonon scattering and up-conversion will further increase, especially if phonon propagation is restricted. This leads to an apparent pumping power-dependent hot-phonon bottleneck effect.

Further considerations about the proposed carrier-phonon dynamics are addressed below.

The mechanism behind the bottleneck effect we have discussed relies on the unique lattice structure and phonon properties of lead-halide perovskites, especially in the organic–inorganic hybrid types. Low-energy optical modes that have a good overlap with acoustic modes need to be introduced to realize a more efficient up-conversion process. This is supposed to be a new and general strategy to achieve long-lived hot carriers in material than opening a large phonon band gap. It is now established that the large band gap is not sufficiently to suppress the decay of hot phonons, especially those via the anti-symmetric Ridley route^60^. Blocking the propagation of acoustic phonons is another aspect to enhance the overall efficiency of up-conversion, since the up-transition probability is usually limited by selection and conservation rules. Materials with a low thermal conductivity are valuable to explore, especially the type with organic–inorganic hybrid compositions.

We also notice that our proposed mechanism has a strong connection to the large polaron effect proposed by Zhu *et al*.^29^ recently. This theory suggests that the freely rotating organic cation can form a long-range polaron along with charge carriers. The large polaron can screen the hot carriers from scattering with LO phonons. This mechanism only relates to the effective strength of Fröhlich interaction in this material (the first kind of blocking mechanism mentioned above) and dominates in the first cooling stage primarily. Instead, the mechanism we proposed here is attempting to explain the bottleneck effect in the following second cooling stage on a timescale of several hundred picoseconds. These two mechanisms are not in contradiction with each other. In fact, the polaron effect suggests a stronger dynamical coupling between the charge carriers and organic cation directly in lead-halide perovskites, which is usually ignored in the conventional understanding. Our study also indicates the rotatable organic cation plays an important role to the acoustic phonon up-conversion in this kind of material. The direct coupling between carriers and organic cation may further facilitate the thermal recycling in the material, since the vibrational energy transferred onto the organic sub-lattice from low-energy modes could reheat charge carriers more efficiently.

In conclusion, we have investigated the bottleneck effect of carrier-phonon cooling in various lead-halide perovskite thin films with different compositions. By comparing both the carrier cooling lifetime and LO emission rate, we conclude that the hybrid perovskites have a stronger phonon bottleneck effect than the pure inorganic types. The phonon relaxation lifetime in FAPbI_3_ is estimated to be more than 10 times longer than that in the CsPbIBr_2_ counterpart under a similar carrier concentration and temperature. The up-conversion of acoustic phonons was proposed as the main factor in the phonon dynamics. In hybrid type perovskites, the low-energy optical phonon modes introduced by the rotatable organic cation show a good band overlap and thermal coupling with the acoustic phonons, allowing an efficient phonon up-conversion. Blocking phonon propagation caused by strong anharmonic scatterings also increases the up-transition probability of low-energy modes. Both aspects mentioned above result in an effective vibrational energy recycling in hybrid lead-halide perovskites and prolong the overall cooling period of the carrier-phonon system. It is valid to note again the poor hot carrier response observed in our MAPbI_3_ sample despite it having a fairly good performance in conventional solar cells. Special attention to the lattice purity is required in the hot carrier applications. The phonon dynamics discussed here are also expected to motivate new explorations of functional hot carrier materials in future.

## Methods

### Sample preparation

#### FAPbI_3_

To prepare 1.2 M HC(NH_2_)_2_PbI_3_ solution, HC(NH_2_)_2_I is mixed with PbI_2_ in dimethylformamide (DMF) at 1:1 mole ratio at room temperature. The solutions with added HI at specific molar ratio (0.10 g of HI solution 1 ml of perovskite solution) were spread on borosilicate glass and spun at 6,500 r.p.m. for 30 s using gas-assisted method^33^. The films were dried on a hot plate at 160 °C for 20 min.

#### MAPbBr_3_

All samples were deposited on borosilicate glass substrates. The substrates were cleaned by 2% Hellmanex detergent, acetone and isopropanol in an ultrasonic bath for 10 min in each cleaning agent followed by UVO treatment for 10 min. All CH_3_NH_3_PbBr_3_ films were fabricated by vapour-assisted method^31^. First, PbBr_2_ solution in DMF with a concentration of 1 M was spin-coated on glass substrate at 2,000 r.p.m. for 60 s. After annealing at 70 °C for 30 min, the film was treated by CH_3_NH_3_Br vapour at 175 °C for 10 min in a closed glass Petri-dish with CH_3_NH_3_Br powder surrounded on a hot plate in a glove box, then rinsed in isopropanol at room temperature, followed by drying in a nitrogen stream.

#### MAPbI_3_

Unless specified otherwise, all materials were purchased from either Alfa Aesar or Sigma-Aldrich and used as received. CH_3_NH_3_I was synthesized by mixing 24 ml CH_3_NH_2_ (33% in ethanol) and 10 ml HI (57% in water) in 100 ml ethanol. After stirring for 2 h, the solvent was removed on a rotary evaporator. The white crystals were dried in a vacuum oven at 60 °C for 24 h. The soda-lime glass substrates were cleaned and then cut into around 1 cm^2^. A 25 μl 45 wt% CH_3_NH_3_PbI_3_ DMF solution, prepared from PbI_2_ and CH_3_NH_3_I in a molar ratio of 1:1, was spread on it, using a spin-coater. For the conventional spin-coating method, the solution was spun at 6,500 r.p.m. for 30 s, while for the gas-assisted method, a 40 psi dry Argon gas stream was blown over the film during spinning at 6,500 r.p.m. for 2 s after the spin-coating commenced. The films were then annealed at 100 °C on a hot plate for 10 min, and then cooled to room temperature on a steel substrate.

#### CsPbIBr_2_

Borosilicate glass was cleaned by sonication in solutions of 2% Hellmanex in deionized water, acetone and isopropanol for 15 min. After drying, the substrate was treated by UV ozone cleaner for 10 min. A dual source thermal evaporation of the two precursors caesium iodide (CsI) and lead bromide (PbBr_2_) was carried out in a thermal evaporation system (Kurt J. Lesker Mini Spectros) integrated in a glove box. CsI and PbBr_2_ were loaded in separate crucible heaters and the sample substrates were fixed on a rotatable substrate holder. After the pressure of the evaporator chamber was pumped down to 10−6 mbar, CsI and PbBr_2_ were then heated to the set temperature of 350 °C and 180 °C, respectively. Once the temperatures were reached, the shutter for each source was opened to commence deposition. The temperature of the substrate holder was kept at 75 °C during the deposition. The deposition rates of CsI and PbBr_2_ were set at 0.21 Å s^−1^ and 0.2 Å s^−1^, respectively, to achieve a molar ratio of 1:1 for the two materials. After the evaporation, the samples were annealed on a hot plate at 250 °C for 10 min in the glove box.

### Ultrafast transient absorption

Femtosecond pump-probe TA experiments were performed on MAPbI_3_, FAPbI_3_, MAPbBr_3_ and CsPbIBr_2_ samples with a TA spectrometer. The laser consisted of a Ti:sapphire mode-locked oscillator that seeded a regenerative amplifier. The output of the amplifier was centred at 800 nm with a repetition rate of 1 kHz and pulse duration of 100 fs, which was then split into pump and probe beamlines. The 400 nm pump pulses were generated using a BBO crystal and was attenuated. The probe beam passed through a delay stage and was used to generate a white light continuum. The probe beam was then detected by a polychromatic-CCD. All of the measurements were performed at room temperature. Lattice heating by the pump laser is shown to be a minor effect in our measuments, see Supplementary Note 1.

### Calculation of phonon band structure

The density functional theory calculations were carried out using Quantum Espresso^61^. The Perdew–Ernzerhof-Burke functional^62^ was used to evaluate the ground state properties, electron and phonon properties. The plane-wave energy cutoff was set to 100 Ry, while a grid of 6 × 6 × 6 was used for k-point sampling. The tolerance for the electronic system was set to 10^−12^ Ry, while that for the phonon calculations was set to 10^−14^ Ry. Density functional perturbation theory was used to predict the phonon energies at a 4 × 4 × 4 grid of **Q** points for FAPbI_3_, MAPbI_3_ and CsPbBr_3_. Lattice constants for the calculations are given in Supplementary Table 1. The phonon band structures were then interpolated from this grid using the interatomic force constants. The phonon band structure for MAPbBr_3_ is extrapolated using the mass approximation approach^63^ with the force constants for MAPbI_3._

### Data availability

The data that support the findings of this study are available from the corresponding author upon request.

## Additional information

**How to cite this article:** Yang, J. *et al*. Acoustic-optical phonon up-conversion and hot-phonon bottleneck in lead-halide perovskites. *Nat. Commun.* **8,** 14120 doi: 10.1038/ncomms14120 (2017).

**Publisher's note:** Springer Nature remains neutral with regard to jurisdictional claims in published maps and institutional affiliations.

## Supplementary Material

Supplementary Information Supplementary Figures, Supplementary Table, Supplementary Note, Supplementary Methods and Supplementary References
