# Supplementary material for: Acoustic-optical phonon up-conversion and hot-phonon bottleneck in lead-halide perovskites
Source: Nat Commun. 2017 Jan 20;8:14120. doi: 10.1038/ncomms14120 (PMC5263885; doi:10.1038/ncomms14120)
Supplement: Supplementary Information — Supplementary Figures, Supplementary Table, Supplementary Note, Supplementary Methods and Supplementary References [file ncomms14120-s1.pdf]

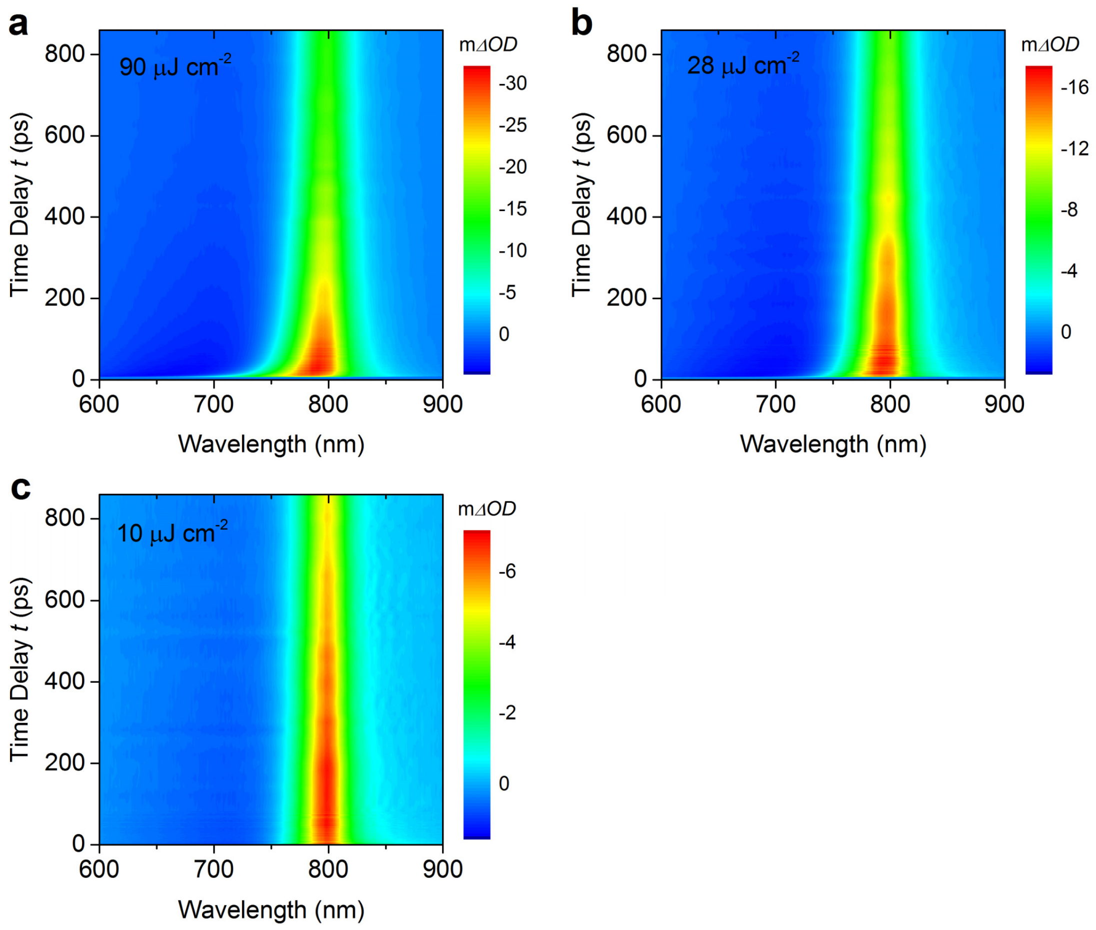

Supplementary Figure 1 Transient absorption (TA) spectrum pumped at 400 nm in the $FAPbI_3$ sample with different excitation intensities and initial carrier concentrations: (a) $N_0 = 4.84 \times 10^{18}$ cm^{-3}; (c) $N_0 = 1.51 \times 10^{18}$ cm^{-3}; (c) $N_0 = 5.38 \times 10^{17}$ cm^{-3}.

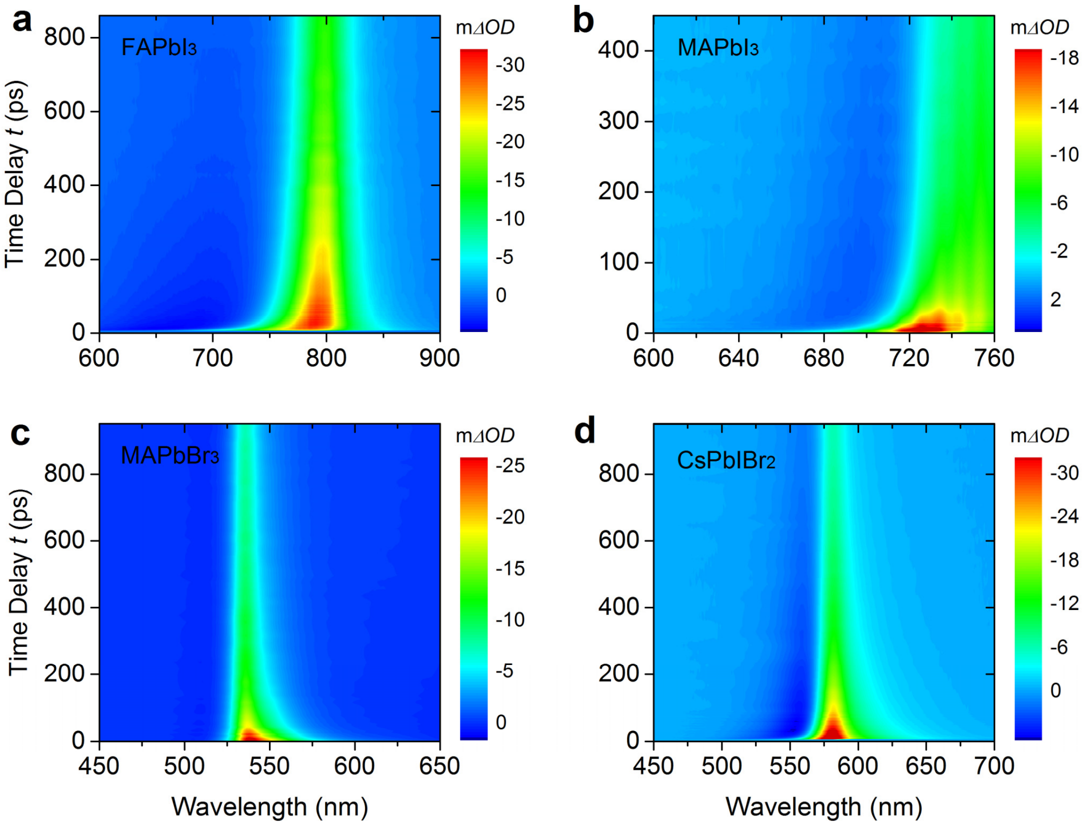

Supplementary Figure 2 Transient absorption (TA) spectrum pumped at 400 nm with an initial carrier concentration around $N_0 = 5 \times 18$ cm^{-3} in four kinds of perovskite samples: (a). $FAPbI_3$; (b) $MAPbI_3$; (c) $MAPbBr_3$; (d) $CsPbIBr_2$

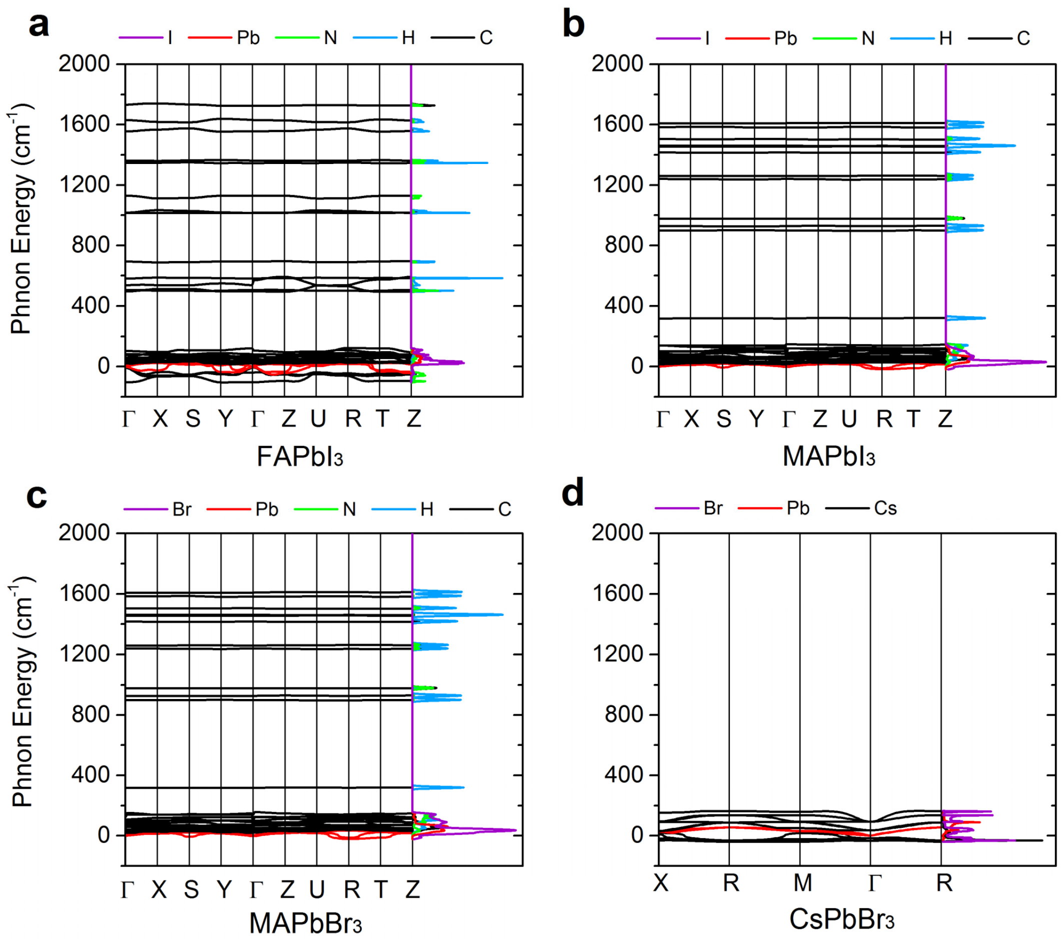

Supplementary Figure 3 Phonon band structure and phonon DOS in (a) $FAPbI_3$; (b) $MAPbI_3$; (c) $MAPbBr_3$; (d) $CsPbBr_3$. The acoustic bands are plotted in red.

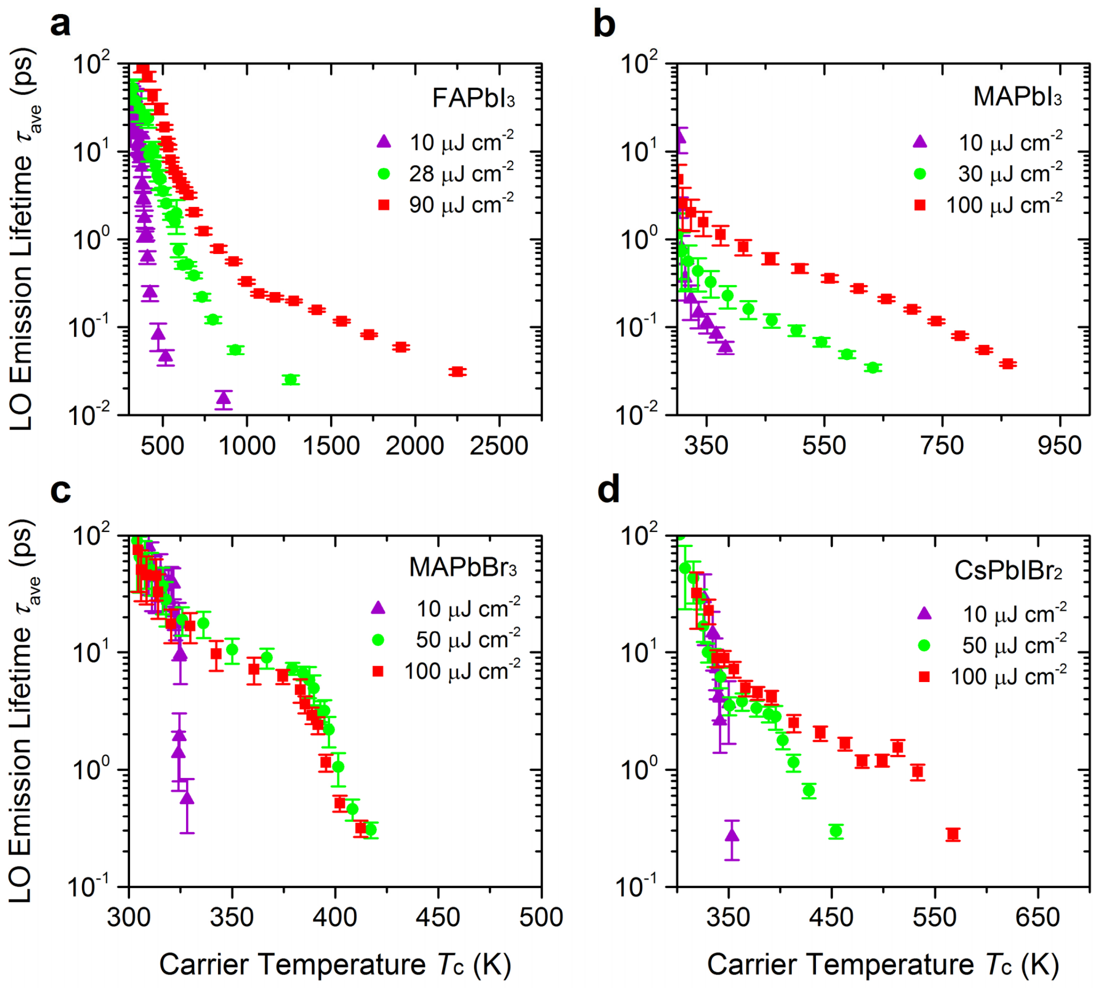

Supplementary Figure 4 LO phonon emission lifetime τ_{ave} versus carrier temperature T_c under different carrier injection levels in: (a) $FAPbI_3$, (b) $MAPbI_3$, (c) $MAPbBr_3$, (d) $CsPbIBr_2$. The error bar shows the standard error (s.e.m) of average τ_{ave}.

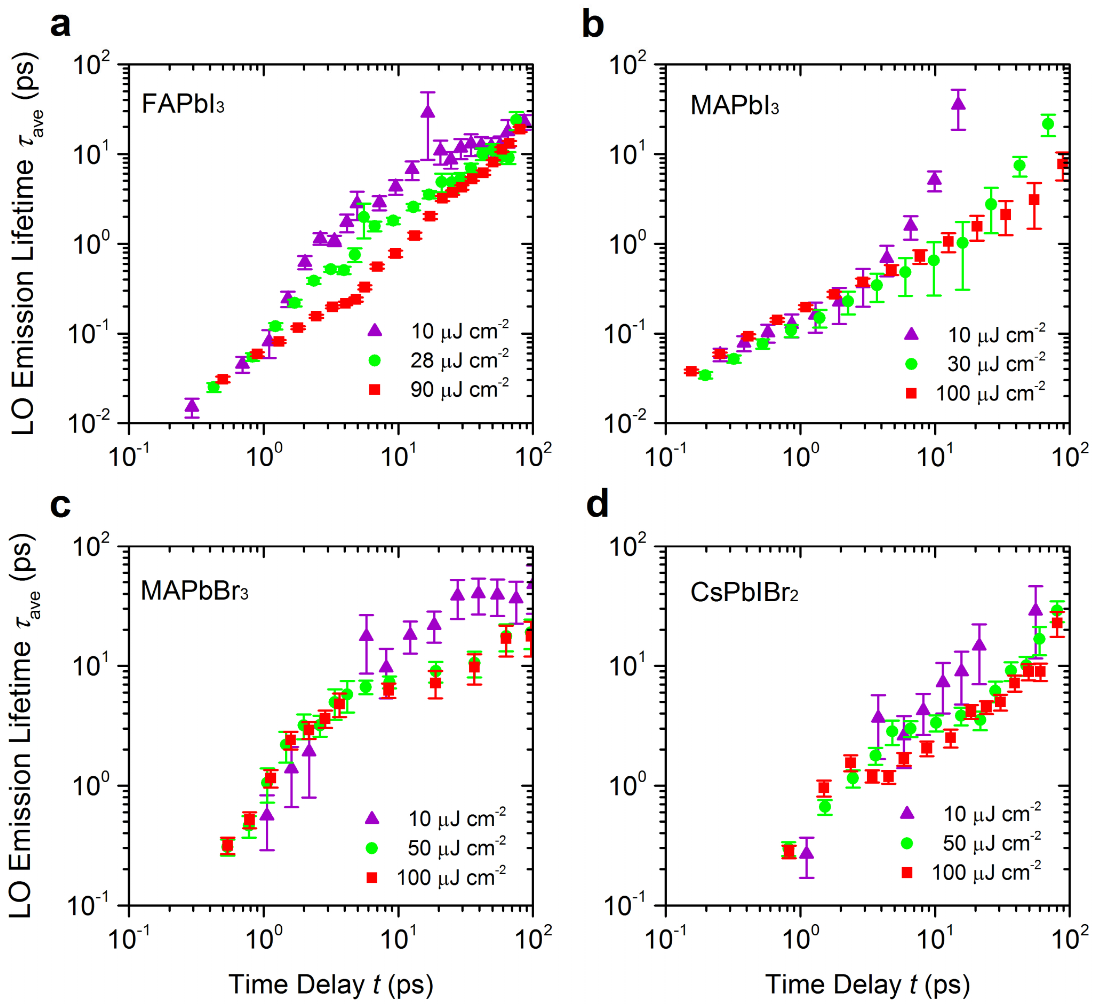

Supplementary Figure 5 LO phonon emission lifetime τ_{ave} varying with time delay t of measurement under different carrier injection levels in: (a) $FAPbI_3$, (b) $MAPbI_3$, (c) $MAPbBr_3$, (d) $CsPbIBr_2$. The error bar shows the standard error (s.e.m) of τ_{ave}.

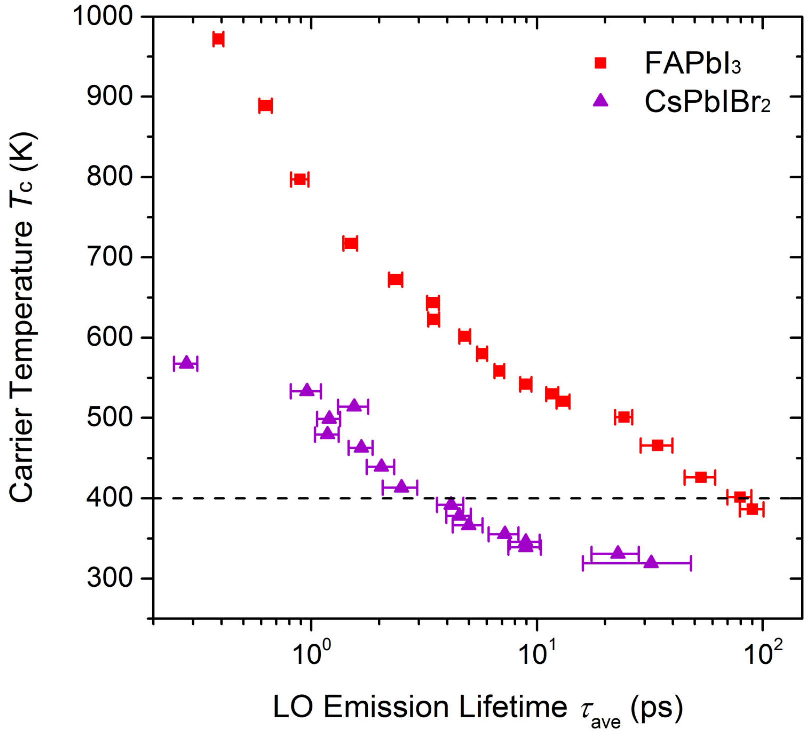

Supplementary Figure 6 Carrier temperature dependent LO phonon emission lifetime τ_{ave} in $FAPbI_3$ and $CsPbIBr_2$ with a similar initial carrier concentration N_0 of around 5×10^{18} cm^{-3}. The emission lifetime in $FAPbI_3$ is around 20 times longer than that in $CsPbIBr_2$ when carrier temperature is around 400 K. The error bar shows the standard error (s.e.m) of τ_{ave}.

Material	$MAPbI_3$	$FAPbI_3$	$CsPbBr_3$
Eg (eV)	1.77	1.4390	1.7310
Lattice constant (Å)	6.29012	a=6.3620<br>b=6.1930<br>c=6.3513	5.9595

Supplementary Table 1. Lattice constants used for phonon band structure calculation in DFT. The phonon band structure for $MAPbBr_3$ is extrapolated using the mass approximation approach with the force constants for $MAPbI_3$.

Supplementary Note 1

All the femtosecond pump-probe TA experiments were performed at room temperature (295 K). Lattice heating by the pump laser is shown to be a minor effect in our measurements. Supposing that all the laser energy that is absorbed by the sample heats the lattice directly, the temperature change can be calculated as below

$$\Delta T_{\mathrm{L}} = \frac{E_{\mathrm{pump}}}{C_{\mathrm{V}} \cdot V_{\mathrm{pump}}} = \frac{F_{\mathrm{pump}} \cdot \alpha_{400}}{C_{\mathrm{V}}} \quad \text{Supplementary Equation 1}$$

In the above equation, C_{V} is the volume heat capacity of perovskites, E_{pump} is the pumping energy, F_{pump} is the corresponding fluence, V_{pump} is excitation volume in the sample and α_{400} is the absorption coefficient of the material at 400 nm. The typical value of C_{V} can be found in supplementary reference 1 which is about 1.25×10^{6} J m^{-3} K at 300 K. With the maximum excitation fluence 100 μJ cm^{-2}, the maximum temperature increase is only about 15-20 K. In practice, considering most of the incident energy will be directly absorbed by the carriers and also given the material's finite thermal conductivity, the actual lattice heating resulting from the laser will be much smaller than the maximum value we deduced above. Therefore, we conclude that direct lattice heating has a negligible impact on our analysis and cannot explain the significant difference in the carrier relaxation in different perovskites samples.

Supplementary Methods

Carrier Injection Level Estimation

The initial photo-generated carrier concentration N_0 is estimated using the following equation.

$$N_0 = \frac{A(\lambda) \times F}{E_{\mathrm{ph}}(\lambda) \times W} \qquad \text{Supplementary Equation 2}$$

, where F is the total incident fluence at excitation wavelength $\lambda = 400$ nm, photon energy $E_{\mathrm{ph}}(\lambda) \approx 4.97 \times 10^{-13}$ μJ, sample thickness $W \approx 300$ nm, $A(\lambda)$ is the overall absorbance of the thin film. When the absorption thickness is much smaller than W, the absorbance can be analytically estimated by

$$A(\lambda) = 1 - R - T = \left(1 - e^{-\alpha(\lambda)\cdot W}\right)\cdot\left(1 - R\right) \qquad \text{Supplementary Equation 3}$$

, where R and T are the reflectance and transmittance respectively, $\alpha(\lambda)$ is the absorption coefficient of material. For the four lead-halide perovskite samples discussed here, the overall absorbance is about $A(400\ \mathrm{nm}) \approx 0.8$ at 400 nm according to previous reports [2–6], where most of the loss in absorption comes from surface reflections. Therefore, N_0 is estimated as 5.38×10^{18} cm^{-3} when $F = 100$ μJ cm^{-2}.

Carrier Temperature Fitting

1. Modified Maxwell-Boltzmann fitting

Bleaching of absorbance reflects the carrier occupation above the band edge after excitation. For carriers distributed across the high-energy region ($E >> \mu_c$), a rigorous Fermi-Dirac distribution defined by carrier temperature T_c and chemical potential μ_c can be approximated by a Maxwell-Boltzmann distribution, as shown in the first term of Eq. Supplementary Equation 4. The second term in that equation represents the photon-induced absorbance (PIA) in a parabolic band approximation. This equation is then fitted to a high-energy tail of bleaching spectrum to extract the carrier temperature. The contribution of PIA must be taken into account in order to further extend the fitting region to higher energies, where the TA spectra usually show a positive ΔOD due to a very low carrier occupation there. The detailed influences of band gap normalization are ignored here since we deal with the high-

energy tail only. This fitting formula can better capture the exponential curvature of a TA spectrum when varying the tail selection scheme. A typical fitting example is shown in Supplementary Figure 7, where the selected tails (see details in the next section) are from 1/3 of the maximum bleaching with a 0.3 eV length.

$$f(E) \approx A_1 \exp\left(-\frac{E}{k_B T_c}\right) + A_2 E^{-1/2} \qquad \text{Supplementary Equation 4}$$

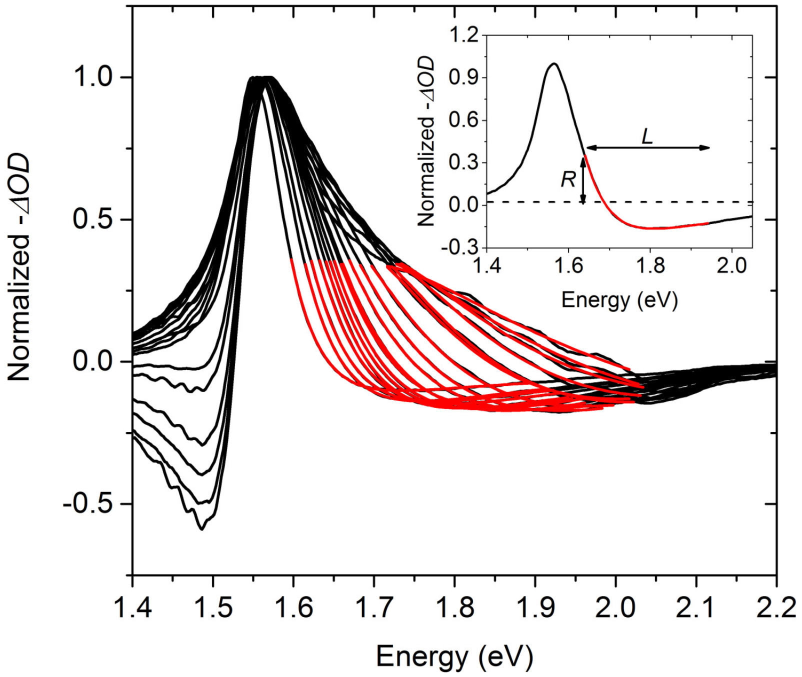

Supplementary Figure 7 High-energy tails on the normalized bleaching spectra of $FAPbI_3$ sample under 28 μJ cm^{-2} when R is around 1/3 and L = 0.3 eV. The black lines are the normalized negative ΔOD ($-\Delta OD$) for different time delays while the fitted results are shown as red curves. The insert shows the definition of tail head ratio R and tail length L on an example TA spectrum.

2. Tail selection and fitting scheme

We always use the following tail selection scheme in all the TA spectra from different samples to ensure comparable results between them:

1. The high-energy tails are always selected from a location whose bleaching intensity has a constant ratio R (e.g. 1/5 initially) to the maximum bleaching, as shown in the insert of Supplementary Figure 7;

2. All tails extend to the high energy region by a constant tail length $L = 0.3$ eV;
3. The modified Maxwell-Boltzmann fitting is applied on the selected tails at each time delay to get carrier temperatures $T_c(t)$;
4. Using the same tail length L, the fitting is then repeated when increasing R from 1/5 to 1/2 linearly (in 10 steps) ;
5. All the fitted $T_c(t)$ at the same time delay t under different R are then averaged as the final result. The error bar showed in the main text and below on the carrier temperature is the corresponding standard error on the mean (s.e.m) at time delay t.

3. Sensitivity analysis

To estimate the global reliability of the fitting results, we also repeat the above fitting procedure with three different tail lengths L that are 0.15 eV, 0.2 eV, and 0.3 eV respectively. All the fitting results are summarized in Supplementary Figure 8. In most of the cases, our fitting scheme shows reasonably stable and reproducible fitting results under different L, especially after a time delay of about 10 ps where most of our discussions and comparisons are made. Most of the large variations are in the first few picoseconds especially when L is small. Large variations in the fitting results also appear when the carrier injection is low, which results from the data quality of the TA spectra.

The adopted fitting scheme only has two input parameters that are the tail head ratio R and tail length L. Based on the fitting results with different R and L in Supplementary Figure 8, the first order sensitivity index $S_R(t)$ of the fitting results on the parameter R is analyzed. This indicates the influence from the starting point of high-energy tails. The first order sensitivity index is defined as

$$
\begin{aligned}
S_R(t) &= \frac{V(E(T_c(t)\,|\,R))}{V(T_c(t))} \\
&= \frac{V(E(T_c(t)\,|\,R))}{E(V(T_c(t)\,|\,R)) + V(E(T_c(t)\,|\,R))}
\end{aligned}
\qquad \text{Supplementary Equation 5}
$$

, where function V calculates the variation while function E takes the mean of the values. The term $V(E(T_c(t)|R))$ denotes the variation of the averaged carrier temperature for a certain time delay t when keeping the R as a constant but varying L. Similarly, $E(V(T_c(t)|R))$ means the average of the variation of the fitted temperatures.

Supplementary Figure 9 shows the statistical distribution of $S_R(t)$ in each sample, while Supplementary Figure 10 shows the distribution of the normalized standard error when varying R. The normalized standard error is defined as a standard error divided by its corresponding mean. All the distributions are counted over the entire time delay period. Roughly speaking, the variation of R contributes about 50% to the variation of the final fitted temperature as shown in Supplementary Figure 9. However, according to Supplementary Figure 10, most of the fitting standard errors are distributed around and below 5% when varying R. This illustrates that the actual numerical impact from the selection of tail starting point is very small. Therefore, we conclude that the numerical results based on our fitting scheme are reasonably stable and insensitive to the choice of the high-energy tail. All the fitting results are reliable and comparable. In the main text and below, we use the fitting results with L = 0.3 eV (the third column in Supplementary Fig. 8) to conduct other analyses.

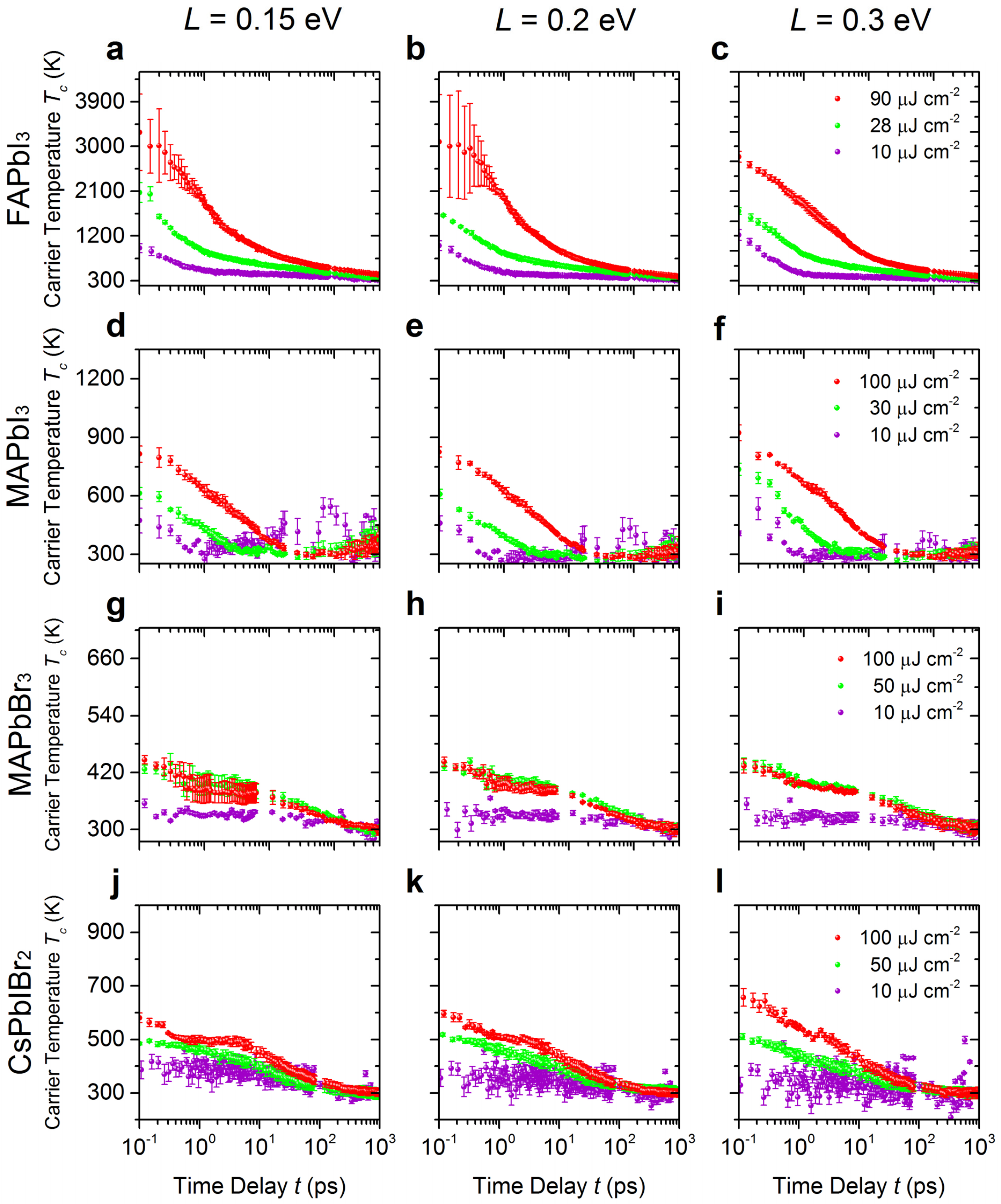

Supplementary Figure 8 Averaged carrier temperature fitting results with different tail length L (0.15 eV, 0.2 eV, 0.3 eV). At the same time delay t, an average fitting result is shown in the graph when the tail head R is changed between 1/2 and 1/5. The error bar shows the corresponding standard error (s.e.m) of the average temperature. In the main text and below, we use the fitting results with L = 0.3 eV to conduct other analyses

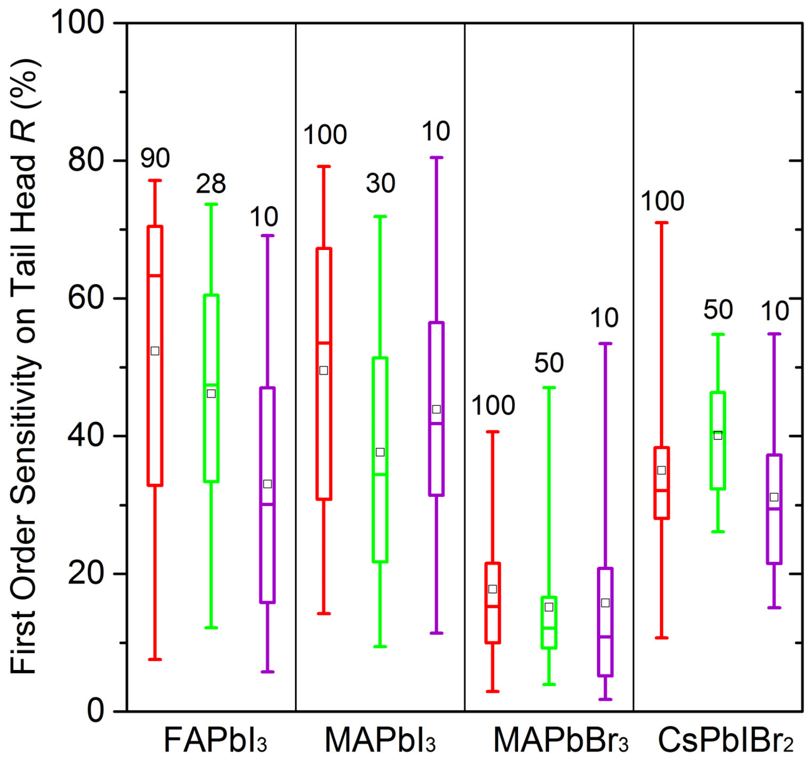

Supplementary Figure 9 Statistical distribution of $S_R(t)$ to the carrier temperature fitting over the entire time delay t is shown in the box plots. The distributions are counted over the entire time delay period. The boxes show the first and third quartiles of the distribution with the median band inside, while the whiskers label 1.5 times of the interquartile range (1.5×IQR). The square dot in the boxes shows the mean of the distribution. Each material has three box plots under different excitation fluences labeled at top of the boxes (unit: μJ cm^{-2}).

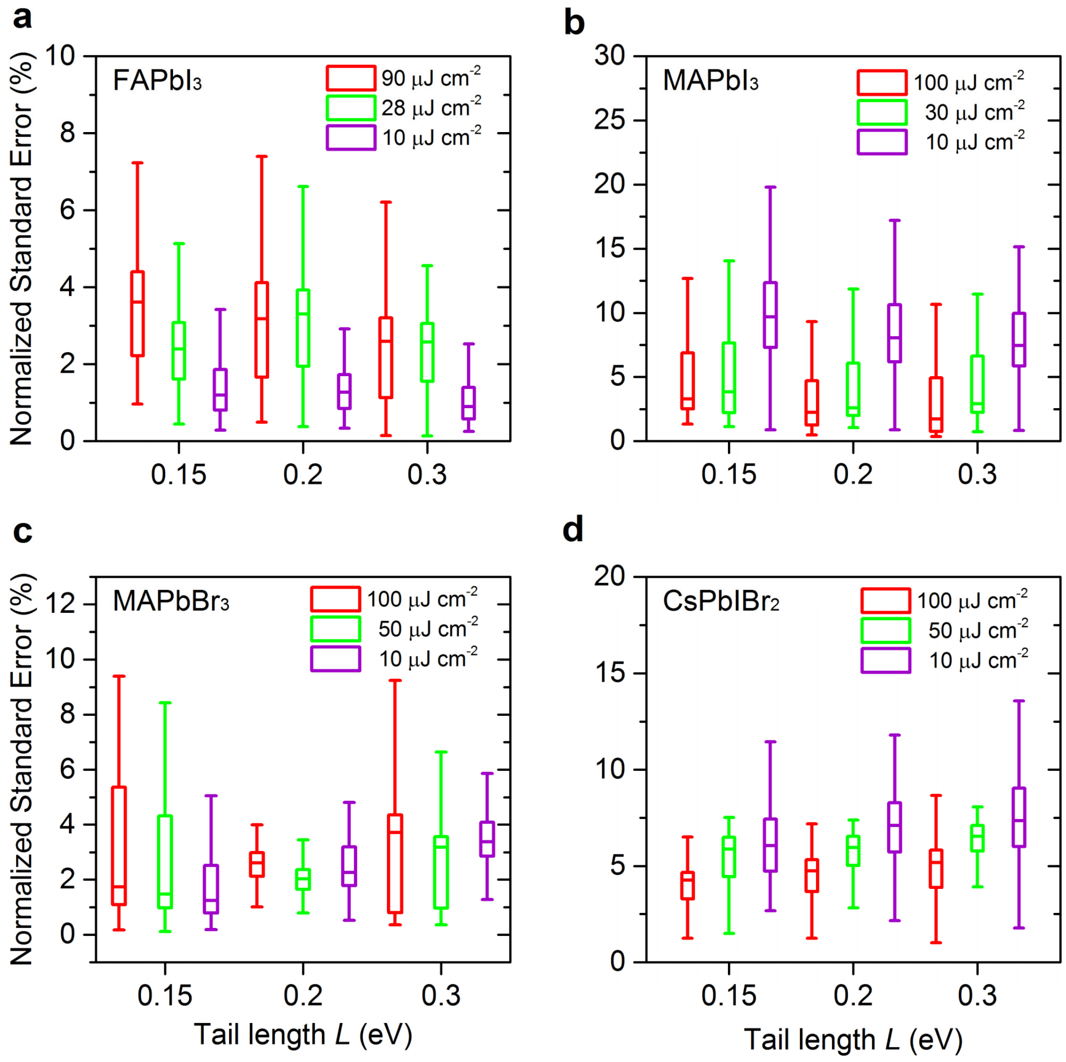

Supplementary Figure 10 Statistical distribution of the normalized fitting standard error when varying the tail head ratio *R* under different tail lengths *L*. The distributions are counted over the entire time delay period. The boxes show the first and third quartiles of the distribution with the median band inside, while the whiskers label 1.5 times of the interquartile range (1.5×IQR).

Supplementary References

1. Qian, X., Gu, X. & Yang, R. Lattice thermal conductivity of organic-inorganic hybrid perovskite $CH_3NH_3PbI_3$. **63902,** 3–8 (2015).
2. Ma, Q., Huang, S., Wen, X., Green, M. A. & Ho-Baillie, A. W. Y. Hole transport layer free inorganic $CsPbIBr_2$ perovskite solar cell by dual source thermal evaporation. *Adv. Energy Mater.* **6,** 1502202 (2016).
3. Kim, J. *et al.* Nucleation and growth control of $HC(NH_2)_2PbI_3$ for planar perovskite solar cell. *J. Phys. Chem. C* **120,** 11262–11267 (2016).
4. Sheng, R. *et al.* Methylammonium lead bromide perovskite-based solar cells by vapour-assisted deposition. *J. Phys. Chem. C* **119,** 3545–3549 (2015).
5. Jiang, Y. *et al.* Time-resolved fluorescence anisotropy study of organic lead halide perovskite. *Sol. Energy Mater. Sol. Cells* **151,** 102–112 (2016).
6. Jiang, Y. *et al.* Temperature dependent optical properties of CH3NH3PbI3 perovskite by spectroscopic ellipsometry. *Appl. Phys. Lett.* **108,** 0–5 (2016).
